# Research progress on the premature ovarian failure caused by cisplatin therapy

**DOI:** 10.3389/fonc.2023.1276310

**Published:** 2023-11-20

**Authors:** Zelin Li, Haodong Qi, Zhengyang Li, Yuxuan Bao, Kangping Yang, Qinghua Min

**Affiliations:** ^1^ The First Clinical Medical College of Nanchang University, Nanchang, China; ^2^ Queen Mary School of Nanchang University, Nanchang, China; ^3^ The Second Clinical Medical College of Nanchang University, Nanchang, China; ^4^ Department of Obstetrics and Gynecology, The First Affiliated Hospital of Nanchang University, Nanchang, China

**Keywords:** cisplatin, premature ovarian failure, apoptosis, protection strategies, research and analysis

## Abstract

Cisplatin is a common anticancer drug able to kill tumor cells, but it causes adverse reactions in the kidney, digestive tract, and other systems. The antitumor effects of cisplatin are mainly due to its ability to bind to the DNA in tumor cells to prevent replication, thereby reducing RNA and protein syntheses, leading to cell damage and death. Cisplatin has a wide range of applications; it can be used to treat cervical, thyroid, ovarian, and other cancers. Cisplatin has a beneficial therapeutic effect, but its therapeutic selectivity is poor. In addition to eliminating diseased target cells, cisplatin can damage normal cells; in women of reproductive age being treated for cancer, cisplatin can lead to ovarian function impairment, premature ovarian failure (POF), and/or infertility. Therefore, reducing the adverse effects of cisplatin on ovarian function is an important topic in clinical research. In this paper, we explore the research progress on the POF caused by cisplatin treatment.

## 1 Introduction

Premature ovarian failure (POF), the premature aging of the ovaries, manifests itself by secondary amenorrhea before the age of 40, with follicle-stimulating hormone levels >40 u/L (1), estradiol levels <20 pg/mL, and low anti-Mullerian hormone values close to 0 ng/mL (2). In addition, ultrasound examinations show no follicles in the ovary and often an atrophic shrunken uterus with a thin endometrium. The etiology of POF is complex and unclear in most patients, but may be related to autoimmune factors, infections, or drugs. Common treatment drugs causing POF include platinum compounds, antimetabolic drugs, and antibiotics. The widely used platinum drugs bind covalently to ovarian DNA, form cross-links between DNA chains, and lead to follicle DNA cleavage during replication, thereby inhibiting follicle DNA transcription and synthesis; however, these toxic effects are not specific to primordial follicles (3). Researchers have suggested that high cisplatin doses can induce hyperactivation of primordial follicles during quiescent states, resulting in loss of ovarian reserve function (4). 2 Cytotoxic mechanisms of cisplatin.

**Figure 1:**
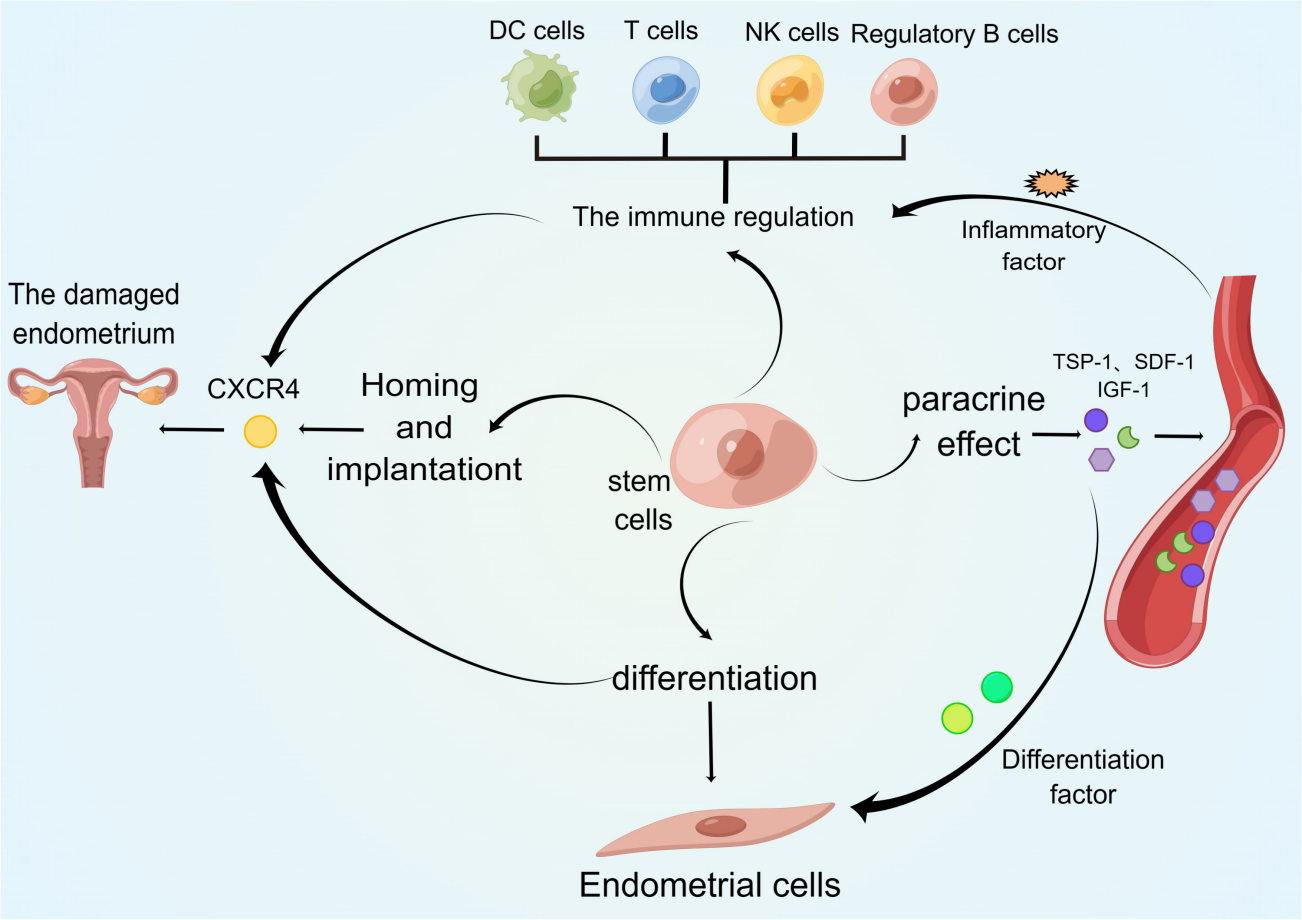
Regulatory cells in premature ovarian failure. This figure shows the major immune cell types and cytokines involved in the pathogenesis of premature ovarian failure.

Cisplatin is a common tumor therapeutic agent available only with a medical prescription due to its toxicity and side effects. Studies have suggested that platinum compounds can be used in cancer therapy, and that these drugs have cytotoxic effects (5). Cisplatin has strong cytotoxic effects that can destroy cancer cells, through its effects on DNA metabolism (**Figure 1**). The drug hydrolyzes in the cell mainly according to the concentration state and the carrier protein channel on the surface of the cell membrane, and then produces binding reaction with other substances. For example, adenosine triphosphate is transported through the endoplasmic reticulum along with cisplatin, glutathione (GSH), and L-cysteine. The fusion of cisplatin with GSH, cysteine (L(+)-Cysteine), and other proteins leads to the depletion of antioxidant reserves in the cytoplasm and causes cellular oxidative stress, which is the major mode of action of cisplatin toxicity. Oxidative stress affects antioxidant enzymes and other molecules, reduces GSH, and increases cisplatin toxicity (6). When cisplatin enters cancer cells, it activates reactive oxygen species pathways, resulting in apoptosis. Cisplatin hydrolysates fuse with DNA bases in the nucleus, and these complexes get identified by the nucleotide excision repair protein (NER), and the mismatch repair (MMR) and Pt-DNA complexes. HMG1 (High Mobility Group 1) and HMG2 (High Mobility Group 2) are two highly conserved nuclear proteins, which play an important role in a variety of biological processes such as DNA repair and apoptosis, and can improve the effect of cisplatin therapy, which includes the following aspects (7): First, the differentiation of in-strand DNA complexes between similar guanines can change the cell cycle activity and induce death. Second, these proteins accelerate the binding of p53 and DNA to induce trans-activation of target genes involved in cell cycle development, DNA repair, and cell apoptosis. Third, they inhibit Pt-DNA complex repair enhancing cisplatin efficacy. NER is an effective enzyme clearing Pt-DNA complexes. If NER is defective, cells become hypersensitive to cisplatin. MMR protein complexes repair DNA damage, but not cisplatin–DNA complexes, and unsuccessful repair initiates cell death. In summary, DNA damage activates multiple cellular signaling pathways that cause cell death. Recent studies have elucidated potential mechanisms of chemotherapy-induced ovarian damage, including DNA damage, oxidative stress, mitochondrial dysfunction, and apoptotic signalling abnormalities induced by cisplatin and other chemotherapeutic agents (8, 9).

## 2 Research progress on the premature ovarian failure induced by cisplatin chemotherapy

### 2.1 Factors influencing premature ovarian failure

The average age of menopause for women is between 50 and 52 years, at which point the ovarian function declines. The main POF inducer is the reproductive system (10). The clinical manifestations of POF include menstrual cycle disorders and infertility. The complex etiology of POF can be divided into two main classes: 1) Idiopathic POF, caused by unknown factors (11); and, 2) POF with clear causes such as genetic and immunological factors. The specific POF mechanisms have remained unclear, and clinical studies have focused on iatrogenic, genetic, and immune system factors.

#### 2.1.1 Genetic factors

Patients with POF have been found to harbor chromosome abnormalities and genetic polymorphisms associated with the development of POF, and among them, 50%–90% of patients present the idiopathic type (12) and show multiple POF-associated genes. The pathogenesis of idiopathic POF includes multiple gene deletions and environmental factors. X chromosome gene mutations have been associated with the occurrence of POF (13), they include mutations in DACH2, FMR1, and BMP15. In addition, some autosomal genes such as FSHR, NR5A1, and BMP15 are associated with POF (14). An association between meiosis gene mutations and ovarian decline has also been demonstrated, underscoring the role of meiosis genes for regulating ovarian function (15).

#### 2.1.2 Immune factors

**Figure 2:**
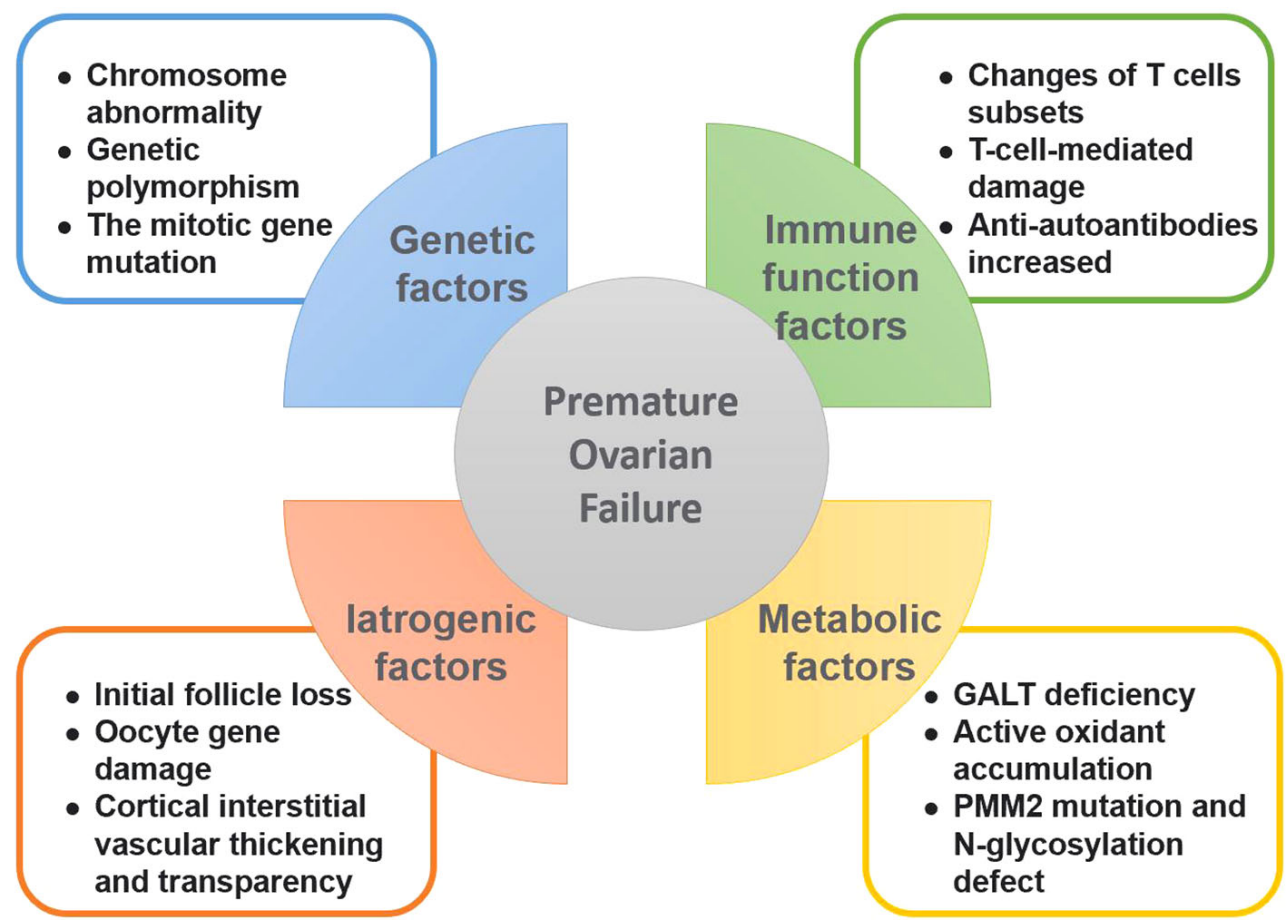
Causative factors of premature ovarian failure. This figure summarizes the various factors leading to premature ovarian failure, including genetic factors, autoimmune factors, iatrogenic factors and metabolic abnormalities.

The ovary is a common autoimmune target in organ-specific and systemic autoimmune diseases, and these can lead to POF, polycystic ovarian syndrome, unexplained infertility, or endometriosis (**Figure 2**). In the case of POF, the autoimmune cause is associated with lymphocytic ovaritis, other autoimmune diseases, and autoantibodies against ovarian antigens. Approximately 20% of patients with primary ovarian insufficiency (POI) have been diagnosed with other autoimmune diseases, often thyroid, adrenal, and pancreatic diseases, and approximately 10% of patients with Addison’s disease develop POI (16). Autoimmune ovarian injury may be associated with a variety of causes, including changes in T cell subsets, T-cell-mediated damage (17), and increased numbers of B cells producing autoantibodies.

#### 2.1.3 Iatrogenic factors

Chemotherapy has multiple effects on ovarian function (18). Initially, during the clinical treatment, transiently induced amenorrhea can induce the growth of follicle groups, and ovarian function decline leads to POF. Once chemotherapy is stopped, the follicular cisterna provides the initial follicles to replenish the growing follicle group that promotes menstruation. However, some initial follicles are absent and lead to premature menopause with the passing of time and ultimately POF. In addition, the drug causes the double-stranded DNA of the oocyte to break causing genetic damage (19) and resulting in oocyte death. Laparoscopic surgery for ovarian cyst causes less tissue trauma, and the postoperative ovarian reserve function indexes and ovarian status are better than those after open surgery (20).

#### 2.1.4 Metabolic factors

Ovarian function is decreased in patients with galactosemia, but the mechanisms responsible for this remain unclear. Patients with galactosemia present a galactose-1-phosphate uridyl transferase deficiency manifested by accumulation of galactose and its metabolites in the body, resulting in ovarian cell swelling and dysfunction (21). In addition, galactose can lead to the accumulation of active oxidants in the body (21), such as methyl glycol, which can block the REDOX cycle of glutathione, thereby destroying ovarian cells. Patients with congenital disorders of glycosylation present PMM2 and N-glycosylation defects that affect the production of follicles and lead to POF (22).

## 3 Effective strategies to reduce cisplatin-induced ovarian toxicity

Cisplatin produces toxicity and side effects, which affect the clinical outcome of patients. Researchers administering cisplatin (5 mg/kg) to rats found marked toxic effects related to the dosage and timing in relation to their sexual cycle (23). The concentration of the medication, the administration route, and the age of patients also affect the actions of cisplatin. During treatment, cisplatin kills many rapidly dividing cells, the endocrine system is greatly affected, and the resulting ovarian dysfunction may be irreversible. Cisplatin combines with DNA to form complexes between and within the chain, affecting cell transcription and causing DNA damage. If left untreated, the drug activates cell-death signaling pathways. Studies have confirmed that the drug can induce ovarian cell death and tissue necrosis (24). In addition, cisplatin activates the non-receptor tyrosine kinase (Abl) and downstream p53 homologous protein in mouse ovarian blast cells, which continuously aggregate to cause oocyte death. Oocyte apoptosis can be reduced by treatment with an Abl activity blocker (18). Cisplatin also activates mitochondrial pathways that release cytochrome C into the cytoplasm leading to caspase pathway activation, which causes a stress response and cell death (25).

## 4 Effective strategies to prevent the POF induced by cisplatin

Due to the significant renal toxicity of cisplatin therapy, pre-treatment hydration therapy must be initiated before the treatment (26): A large infusion needs to be given 2–16 hours before and at least 6 hours after the cisplatin administration. In addition, patients need to drink plenty water and undergo diuretic treatments to ensure a daily urine volume within 2000–3000 mL. Changes in blood potassium and magnesium concentrations also need monitoring to avoid kidney function injury and cardiac burden. The discussion on side effects mitigation strategies was removed to align with the focus on POF.

For gastrointestinal toxicity causing nausea and vomiting, antiemesis drugs can be used before the medication, a four-drug combination regimen consisting of NK1 receptor antagonist, 5-HT3 receptor antagonist, dexamethasone, and olanzapine has been recommended by the American Society of Clinical Oncology guidelines (27).

For hematological toxicity, granulocyte colony stimulating factor (G-CSF), recombinant human platelet growth factor, erythropoietin (EPO) and other drugs can be used to treat the symptoms, the chemotherapy doses can be adjusted, or blood transfusions may be required (28).

The neurotoxicity caused by cisplatin treatment cannot be avoided. Some neuroprotectants may be effective, such as α-lipoic acid (29), but their value needs to be validated by clinical studies.

## 5 Drug protection of ovarian function

### 5.1 Hormone therapy

An efficient hormone therapy would allow for completion of adolescent development and alleviation of hormone deficiencies and sequelae. Clinical treatment for individual patients should consider the advantages and disadvantages of drugs (**Table 1**). Common secondary characteristics of primary POF are lacking and the bone mineral density is decreased. A common regimen includes estradiol treatment, with small initial doses and gradual progression, and the development of secondary sex characteristics can be observed (35). Progesterone protects the endometrium. The purpose of a subsequent treatment is to ensure continual effects and prevent infertility. Hormonal replacement therapy is used in patients with secondary amenorrhea and complete development to decrease cardiovascular problems.

**Table 1:** Some medications to protect ovarian function.

Intervention drug	Dosage	Sample capacity	Clinical result	Ref.
Estradiol with levonorgestrel	estradiol 2 mg daily, with levonorgestrel 75 mcg for 12 days a month	Control group n=6, treatment group n=6	A significantly increased bone density at the lumbar spine	(30)
Conjugated equine estrogen with medroxyprogesterone acetate	0.625 mg conjugated equine estrogen daily plus 5 mg medroxyprogesterone acetate	Control group n=9, treatment group n=9	Endothelial function was restored within 6 months	(31)
leuprorelin	Leprerelin 3.75 mg was injected subcutaneously every 28 days until the end of chemotherapy	Control group n=50, treatment group n=55	Women treated with GnRH agonists had better relapse-free survival	(32)
Progynova(Bayer) with Abbott (Valerate estradiol)	2 mg daily valerate estradiol adding 10 mg daily dihydrogesterone	Control group n=26, treatment group n=26	Decreased levels of circulating follicle stimulating hormone	(33)
Goserelin	Goserelin 3-4 weekly	Control group n=112, treatment group n=109	The incidence of POI decreased by 16.3% between 12 and 24 months	(34)

### 5.2 GnRH analogues

Oocytes divide and proliferate often and are thereby prone to genetic damage. Cisplatin drugs damage mature follicles and deplete the primordial follicles. GnRH analogues can block ovarian activity, maintaining the tissues and making them less sensitive to cisplatin cytotoxicity. In addition, GnRH agonists can reduce blood flow and the concentration of local chemotherapeutic agent and alleviate ovarian function injuries. GnRH agonists can stimulate gonadotropin bursts, causing the primordial follicles to develop into immature follicles. Chemotherapy drugs act on actively dividing cells, and as a result, the ovaries become natural targets for these drugs. For this reason, GnRH agonists are clinically recommended 2 weeks before treatment. Studies have validated the protective effect of GnRH to preserve the ovarian function. In a study, premenopausal women aged between 20 and 40 years who received adjuvant chemotherapy for breast cancer from January 2002 to April 2012 were classified into two groups: Women in one group were treated with GnRH agonists for ovarian protection during chemotherapy, and those in the other group were not administered GnRH. The survival analysis using stratified Cox regression showed that women treated with concurrent GnRH agonists had better recurrence-free survival (adjusted hazard ratio, 0.21; P = 0.009; unadjusted hazard ratio, 0.33; P = 0.034) (32). Thus, GnRH agonists during chemotherapy maintain the therapeutic effect of the chemotherapeutic agent and protect the ovarian function. However, recent studies have questioned the protective effect of GnRH agonists on ovarian function during chemotherapy, indicating that GnRH agonists may not prevent chemotherapy-induced ovarian damage (36) Another study by Turan et al. suggested that there is insufficient evidence to support the biologic basis and clinical utility of GnRH agonists for fertility preservation in cancer patients (37). The utility of GnRH agonists for fertility preservation remains controversial and requires further research.

### 5.3 Traditional Chinese medicine treatment

Traditional Chinese medical treatments for kidney can reduce the side effects of chemotherapeutic drugs, and the selection of drugs that promote adequate blood circulation in patients is done mainly to improve the function of the ovaries and promote the correct functioning of the uterus (38). Other Chinese remedies include deer fetal ointment to relieve the symptoms of maladaptation and to regulate the endocrine system. However, the research in this area is ongoing and in the animal experimental stages; therefore, clinical application is not available.

### 5.4 Estrogen therapy

Nierestrol can reduce the end-metabolism of lipid peroxides *in vivo (* 39). The decrease in the hydroxyl radical levels and the increase in SOD activity can help protect the ovarian function and reduce the toxic effect of chemotherapeutic drugs.

## 6 Conclusion

Platinum anticancer drugs such of cisplatin are cell cycle non-specific drugs that act on the cellular DNA. During clinical treatments, these drugs affect ovarian function and cause ovarian impairment that can lead to POF. POF is the main factor affecting female infertility. However, POF can progress without clear manifestations, and clinical diagnoses are mostly made during later stages of the disease. POF diversity and the genetic heterogeneity in women may contribute to the regulation of ovarian function. Therefore, understanding the genetic mechanisms of POF may lead to early detection and improvements in treatments for the condition. Knowledge of the chemotherapy-related effects can be used to prevent disease occurrences and provide a reliable basis for clinical treatments.

## Author contributions

ZL: Writing – original draft, Writing – review & editing. HQ: Writing – original draft. ZL: Writing – original draft. YB: Writing – review & editing. KY: Writing – review & editing. QM: Supervision, Writing – review & editing.

## References

[1] Cohen J Chabbert-Buffet N Darai E. Diminished ovarian reserve, premature ovarian failure, poor ovarian responder–a plea for universal definitions. J Assist Reprod Genet (2015) 32(12):1709–12. doi: 10.1007/s10815-015-0595-y PMC4681731 26463876

[2] Ceccarelli F Orefice V Perrone G Pirone C Perricone C Truglia S. Premature ovarian failure in patients affected by systemic lupus erythematosus: a cross-sectional study. Clin Exp Rheumatol (2020) 38(3):450–4. 32083540

[3] Massacesi C Bascioni R Cellerino R Scartozzi M Bracci R Alessandroni P. Cisplatin, epirubicin and cyclophosphamide (PEC) in the treatment of advanced ovarian cancer. J Exp Clin Cancer Res (2000) 19(1):13–6. 10840930

[4] Xie Y Li S Zhou L Lin H Jiao X Qiu Q. Rapamycin preserves the primordial follicle pool during cisplatin treatment in vitro and in vivo. Mol Reprod Dev (2020) 87(4):442–53. doi: 10.1002/mrd.23330 32112509

[5] Song M Cui M Liu K. Therapeutic strategies to overcome cisplatin resistance in ovarian cancer. Eur J Med Chem (2022) 232:114205. doi: 10.1016/j.ejmech.2022.114205 35217497

[6] Ahmed EA Omar HM Aelghaffar S Kh Abd Ragb SMM Nasser AY. The antioxidant activity of vitamin C, DPPD and L-cysteine against Cisplatin-induced testicular oxidative damage in rats. Food Chem Toxicol (2011) 49(5):1115–21. doi: 10.1016/j.fct.2011.02.002 21310208

[7] Dilruba S Kalayda GV. Platinum-based drugs: past, present and future. Cancer Chemother Pharmacol (2016) 77(6):1103–24. doi: 10.1007/s00280-016-2976-z 26886018

[8] Bedoschi GM Navarro PA Oktay KH. Novel insights into the pathophysiology of chemotherapy-induced damage to the ovary. Panminerva Med (2019) 61(1). doi: 10.23736/S0031-0808.18.03494-8 29962184

[9] Bedoschi G Navarro PA Oktay K. Chemotherapy-induced damage to ovary: mechanisms and clinical impact. Future Oncol (2016) 12(20):2333–44. doi: 10.2217/fon-2016-0176 PMC5066134 27402553

[10] Davis SR Baber RJ. Treating menopause - MHT and beyond. Nat Rev Endocrinol (2022) 18(8):490–502. doi: 10.1038/s41574-022-00685-4 35624141

[11] Shah D Nagarajan N. Premature menopause - Meeting the needs. Post Reprod Health (2014) 20(2):62–8. doi: 10.1177/2053369114531909 24879744

[12] Cordts EB Christofolini DM Santos AAD Bianco B Barbosa CP. Genetic aspects of premature ovarian failure: a literature review. Arch Gynecol Obstet (2011) 283(3):635–43. doi: 10.1007/s00404-010-1815-4 21188402

[13] Beke A Piko H Haltrich I Csomor J Matolcsy A Fekete G. Molecular cytogenetic analysis of Xq critical regions in premature ovarian failure. Mol Cytogenet (2013) 6(1):62. doi: 10.1186/1755-8166-6-62 24359613 PMC3914679

[14] Persani L Rossetti R Cacciatore C. Genes involved in human premature ovarian failure. J Mol Endocrinol (2010) 45(5):257–79. doi: 10.1677/JME-10-0070 20668067

[15] Caburet S Arboleda VA Llano E Overbeek PA Barbero JL Oka K. Mutant cohesin in premature ovarian failure. N Engl J Med (2014) 370(10):943–9. doi: 10.1056/NEJMoa1309635 PMC4068824 24597867

[16] Ayesha Jha V Goswami D. Premature ovarian failure: an association with autoimmune diseases. J Clin Diagn Res (2016) 10(10):Qc10–qc12. 10.7860/JCDR/2016/22027.8671 PMC5121739 27891401

[17] Gao H Gao L Wang W. Advances in the cellular immunological pathogenesis and related treatment of primary ovarian insufficiency. Am J Reprod Immunol (2022) 88(5):e13622. doi: 10.1111/aji.13622 36087022

[18] Morgan S Anderson RA Gourley C Wallace WH Spears N. How do chemotherapeutic agents damage the ovary? Hum Reprod Update (2012) 18(5):525–35. doi: 10.1093/humupd/dms022 22647504

[19] Oktay K Turan V Titus S Stobezki R Liu L. BRCA mutations, DNA repair deficiency, and ovarian aging. Biol Reprod (2015) 93(3):67. 26224004 10.1095/biolreprod.115.132290 PMC4710189

[20] Leetanaporn R Tintara H. A comparative study of outcome of laparoscopic salpingo-oophorectomy versus open salpingo-oophorectomy. J Obstet Gynaecol Res (1996) 22(1):79–83. doi: 10.1111/j.1447-0756.1996.tb00941.x 8624898

[21] Thakur M Feldman G Puscheck EE. Primary ovarian insufficiency in classic galactosemia: current understanding and future research opportunities. J Assist Reprod Genet (2018) 35(1):3–16. doi: 10.1007/s10815-017-1039-7 28932969 PMC5758462

[22] Wolthuis DF Janssen MC Cassiman D Lefeber DJ Morava E. Defining the phenotype and diagnostic considerations in adults with congenital disorders of N-linked glycosylation. Expert Rev Mol Diagn (2014) 14(2):217–24. doi: 10.1586/14737159.2014.890052 24524732

[23] Yucebilgin MS Terek MC Ozsaran A Akercan F Zekioglu O Isik E. Effect of chemotherapy on primordial follicular reserve of rat: an animal model of premature ovarian failure and infertility. Aust N Z J Obstet Gynaecol (2004) 44(1):6–9. doi: 10.1111/j.1479-828X.2004.00143.x 15089860

[24] Rossi V Lispi M Longobardi S Mattei M Di Rella F Salustri A. LH prevents cisplatin-induced apoptosis in oocytes and preserves female fertility in mouse. Cell Death Differ (2017) 24(1):72–82. doi: 10.1038/cdd.2016.97 27689876 PMC5260508

[25] Clark JS Faisal A Baliga R Nagamine Y Arany I. Cisplatin induces apoptosis through the ERK-p66shc pathway in renal proximal tubule cells. Cancer Lett (2010) 297(2):165–70. doi: 10.1016/j.canlet.2010.05.007 20547441

[26] Oka T Kimura T Suzumura T Yoshimoto N Nakai T Yamamoto N. Magnesium supplementation and high volume hydration reduce the renal toxicity caused by cisplatin-based chemotherapy in patients with lung cancer: a toxicity study. BMC Pharmacol Toxicol (2014) 15:70. doi: 10.1186/2050-6511-15-70 25472655 PMC4272804

[27] Kris MG Hesketh PJ Somerfield MR Feyer P Clark-Snow R Koeller JM. American Society of Clinical Oncology guideline for antiemetics in oncology: update 2006. J Clin Oncol (2006) 24(18):2932–47. doi: 10.1200/JCO.2006.06.9591 16717289

[28] Koinis F Nintos G Georgoulias V Kotsakis A. Therapeutic strategies for chemotherapy-induced neutropenia in patients with solid tumors. Expert Opin Pharmacother (2015) 16(10):1505–19. doi: 10.1517/14656566.2015.1055248 26077189

[29] Kim KH Lee B Kim Y-R Kim M-A Ryu N Da Jung J. Evaluating protective and therapeutic effects of alpha-lipoic acid on cisplatin-induced ototoxicity. Cell Death Dis (2018) 9(8):827. doi: 10.1038/s41419-018-0888-z 30068942 PMC6070527

[30] Cartwright B Robinson J Seed PT Fogelman I Rymer J. Hormone replacement therapy versus the combined oral contraceptive pill in premature ovarian failure: A randomized controlled trial of the effects on bone mineral density. J Clin Endocrinol Metab (2016) 101(9):3497–505. doi: 10.1210/jc.2015-4063 27340881

[31] Kalantaridou SN Naka KK Papanikolaou E Kazakos N Kravariti M Calis KA. Impaired endothelial function in young women with premature ovarian failure: normalization with hormone therapy. J Clin Endocrinol Metab (2004) 89(8):3907–13. doi: 10.1210/jc.2004-0015 15292326

[32] Kim J Kim M Lee JH Lee H Lee SK Bae SY. Ovarian function preservation with GnRH agonist in young breast cancer patients: does it impede the effect of adjuvant chemotherapy? Breast (2014) 23(5):670–5. doi: 10.1016/j.breast.2014.07.005 25088482

[33] Pinelli S Artini PG Basile S Obino MER Sergiampietri C Giannarelli D. Estrogen treatment in infertile women with premature ovarian insufficiency in transitional phase: a retrospective analysis. J Assisted Reprod Genet (2017) 35(3):475–82. 10.1007/s10815-017-1096-y PMC5904066 29204869

[34] Leonard RCF Adamson DJA Bertelli G Mansi J Yellowlees A Dunlop J. GnRH agonist for protection against ovarian toxicity during chemotherapy for early breast cancer: the Anglo Celtic Group OPTION trial. Ann Oncol (2017) 28(8):1811–6. doi: 10.1093/annonc/mdx184 28472240

[35] Costa GPO Ferreira-Filho ES Simoes RDS Soares-Junior JM Baracat EC Maciel GAR. Impact of hormone therapy on the bone density of women with premature ovarian insufficiency: A systematic review. Maturitas (2023) 167:105–12. doi: 10.1016/j.maturitas.2022.09.011 36368093

[36] Adams HJA Kwee TC. The predictive value of interim FDG-PET in early-stage Hodgkin lymphoma is not well established. Ann Oncol (2018) 29(2):510–2. doi: 10.1093/annonc/mdx644 29045539

[37] Turan V Bedoschi G Rodriguez-Wallberg K Sonmezer M Pacheco FS Oktem O. Utility of gonadotropin-releasing hormone agonists for fertility preservation: lack of biologic basis and the need to prioritize proven methods. J Clin Oncol (2019) 37(1):84–6. doi: 10.1200/JCO.18.00420 30407897

[38] Li M Xiao Y-B Wei L Liu Q Liu P-Y Yao J-F. Beneficial effects of traditional Chinese medicine in the treatment of premature ovarian failure. Evid Based Complement Alternat Med (2022) 2022:5413504. doi: 10.1155/2022/5413504 36471694 PMC9719426

[39] Hoang VT Nguyen H-P Nguyen VN Hoang DM Nguyen T-ST Thanh LN. Adipose-derived mesenchymal stem cell therapy for the management of female sexual dysfunction: Literature reviews and study design of a clinical trial. Front Cell Dev Biol (2022) 10:956274. doi: 10.3389/fcell.2022.956274 36247008 PMC9554747

